# A Demonstration of Hypnotism

**Published:** 1893-04

**Authors:** 


### Editorial, Etc..

### A DEMONSTRATION OF HYPNOTISM.

AT THE MEETING OF THE ASSOCIATION OF DENTAL SURGEONS, OF BALTIMORE, Wm. Lee Howard, M. D., was invited to speak upon the value of hypnotism to the dental profession. We give a brief synopsis of Dr. Howard's practical talk:

*Gentlemen*: It is a very difficult task for me in a few minutes talk to go any further than that of giving some practical illustrations and statements of the total anæsthesia that can be produced by the physical force, misnamed hypnotism. Its interest to you solely is that by this force you can extract teeth and perform any dental operations painlessly and safely without the use of the various anæsthetics now in use. From our point of view hypnotism is soon to revolutionize the present treatment of morphinism, alcoholism, insomnia, and the various nervous diseases. As interesting as it would be for me to explain to you the inhibitory action produced by hypnotism, its various conditions, and its universal use at present in the large hospitals in Europe, I will confine myself to facts. Your worthy president has had a practical illustration of its usefulness in the extraction of teeth, and with his permission I will quote his case, and then proceed to place some one in this room in a condition of total anæsthesia. The following is Dr. Twilley's case:

Dr. Twilley, with several professional friends, came to my office one evening to test the total anæsthesia produced in a patient under hypnotic influence, who had suffered from severe toothache for some time. Placing the patient in a chair I immediately put him into a deep catalepsy. Then I said, "Dr. Twilley, go ahead and extract the tooth." He approached the patient and said, "Open your mouth, please." Not getting any response he attempted to part the jaws but found them rigidly fixed. After several of the other gentlemen had tried to get some sign of consciousness without any success, I said in a decided tone of voice: "Now, open your mouth wide." He immediately obeyed. Then Dr. Twilley taking his dental forceps went to work. No gentle hand manipulated those forceps, and if ever a man attempted to torture another, Dr. Twilley certainly deserves to be entitled to that credit. After the tooth was extracted I brought the subject out from the hypnotic state and asked him if he still desired the tooth out. "No, Doctor, I think not now, it does not pain me." "Well, here it is," said Doctor Twilley holding the big molar up still grasped in the forceps. The look of surprise shown by the patient was something never to be forgotten.

In acute inflammatory rheumatism I have several times attempted to suggest the absence of pain but with very unsatisfactory results. In another case, Dr. Atkinson, Dean of the Maryland University, Drs. Finney and Baltzell of the Johns Hopkins University, Prof. Coale, Drs. Wise, Van Bibber and Kinley, made all possible tests as regards her being in a condition of total anæsthesia. The reflexes as is usual in such cases, were exaggerated, unless I desire to totally abolish them.

One of the first things I do when I wish to operate, or produce a normal sleep, is to bring down the heart beats and rapid respirations at once to the normal conditions. One always finds rapid respiration and pulse when the subjects are first hypnotized. *Never neglect to put them in a normal condition before leaving the patient.*

In hypnotism we avoid all the disagreeable sequellæ found in the use of the various gases. I claim that almost all persons who are willing to submit to a dental operation can be hypnotized. For if they have made up their minds to take gas, or suffer, they certainly will give you their attention sufficiently for you to produce a state of hypnosis. At some other time I will gladly go into the physiological and psycological explanation of the science of hypnotism. Now let us get at the practical part of the subject.

Dr. Howard then proceeded by using some of the younger members of the society to prove that he could make them see whatever he suggested.

For reasons which will appear obvious, he did not care to place any of the members in a complete hypnosis; but walking up to a large German waiter, whom he had seen for the first time that evening, said to him, "Close your eyes!" "They are fastly closed now, you can't open them." "Try as hard as you can, you can't open them." The attempt proved useless, as the eyes seemed fastly tightened. Then Dr. Howard asking several gentlemen to take out their watches and count the pulse, ran up the heart's beats, from seventy-five to one hundred and forty, and down again to sixty, all in less than two minutes. This he did several times. After placing the heart in its normal condition, he told the man he was a log of wood and falling down. Prone on the floor fell the man, and every method taken to bring forth some signs of consciousness. Big scarf pins were thrust into his body and through the sensitive nerve endings without any more effect than would have been produced had they been thrust through the table cloth. It is interesting to note that when Dr. Howard finished, his pulse was up to one hundred and fifty beats per minute, respiration in proportion.

UNDER the suggestive title, "The Revival of Witchcraft," Dr. Ernest Hart, the eminent English physician, contributes to the February number of the Nineteenth Century a most interesting paper describing recent tests made by him touching the genuineness of the hypnotic experiments conducted by Dr. Luys at the celebrated Hospital of La Charite in Paris. As a result of two weeks of investigation he affirms that he found "the whole series of performances to be based upon a fraud," and that he "succeeded in reproducing the phenomena without employing any occult means or invoking any new powers of mind or body." Dr. Hart recites minutely all the particulars of his investigations. His experiments embraced three classes of cases, the subjects in each were the same employed by the French physicians, and were all conducted in the presence of competent and credible witnesses, some of whom were firm believers in the marvels of the "new hypnotism." as its disciples are fond of calling it

Dr. Hart does not charge that all the subjects were altogether imposters. Two of them "were pronounced hysterics and trained hypnotics, and mingled pathological conditions and an artificially induced state of partial automatism with their abundant frauds." Others appeared from first to last to be acting a part. Nor does he dispute the power or danger of hypnotic suggestion. The evils and abuses to which it may give rise he emphasizes very strongly. As a medical agency he sees little or nothing of value in hypnotism, its sphere, according to the best authorities, "being limited to the relief of functional disorder and symptoms in hysterical patients." In fine, he holds that "no new faculty was ever yet developed in any of these hypnotics, and that claims of this character "are for the most part demonstrable frauds, or, perhaps, in a few cases, self-deception.

